# Perturbation of Defense Pathways by Low-Dose Arsenic Exposure in Zebrafish Embryos

**DOI:** 10.1289/ehp.0900555

**Published:** 2009-02-22

**Authors:** Carolyn J. Mattingly, Thomas H. Hampton, Kimberly M. Brothers, Nina E. Griffin, Antonio Planchart

**Affiliations:** 1 Mount Desert Island Biological Laboratory, Salisbury Cove, Maine, USA; 2 Center for Environmental Health Sciences, Dartmouth Medical School, Hanover, New Hampshire, USA; 3 University of Maine, Orono, Maine, USA

**Keywords:** arsenic, Comparative Toxicogenomics Database, embryonic development, gene regulatory networks, immunity, microarray, toxicogenomics, transcriptional profiling, zebrafish

## Abstract

**Background:**

Exposure to arsenic is a critical risk factor in the complex interplay among genetics, the environment, and human disease. Despite the potential for *in utero* exposure, the mechanism of arsenic action on vertebrate development and disease is unknown.

**Objectives:**

The objective of this study was to identify genes and gene networks perturbed by arsenic during development in order to enhance understanding of the molecular mechanisms of arsenic action.

**Methods:**

We exposed zebrafish embryos at 0.25–1.25 hr postfertilization to 10 or 100 ppb arsenic for 24 or 48 hr. We then used total RNA to interrogate genome microarrays and to test levels of gene expression changes by quantitative real-time polymerase chain reaction (QPCR). Computational analysis was used to identify gene expression networks perturbed by arsenic during vertebrate development.

**Results:**

We identified a set of 99 genes that responded to low levels of arsenic. Nineteen of these genes were predicted to function in a common regulatory network that was significantly associated with immune response and cancer (*p* < 10^−41^). Arsenic-mediated expression changes were validated by QPCR.

**Conclusions:**

In this study we demonstrated that arsenic significantly down-regulates expression levels of multiple genes potentially critical for regulating the establishment of an immune response. The data also provide molecular evidence consistent with phenotypic observations reported in other model systems. Additional mechanistic studies will help explain molecular events regulating early stages of the immune system and long-term consequences of arsenic-mediated perturbation of this system during development.

Arsenic represents a global environmental health threat and a known human carcinogen. More than 100,000 individuals in New England are exposed to arsenic levels in drinking water that exceed federal standards [U.S. Environmental Protection Agency (EPA)], and 500 million people worldwide are at risk of exposure from contaminated groundwater (Mead 2005). Studies linking arsenic to adverse human health effects such as lung, bladder, and skin cancer were instrumental in motivating recent U.S. policy changes lowering maximum allowable limits in drinking water from 50 ppb to 10 ppb (U.S. EPA 2006). However, the effects of exposure to low levels of arsenic also remain unclear.

Exposure to very low levels of arsenic (< 10 ppb) is linked to adverse biological effects, including endocrine disruption and alteration in cell cycle kinetics, cell signaling, and the proliferative response (Rossman et al. 2004). Arsenic levels commonly found in contaminated drinking water in the U.S. elicit changes in gene expression profiles in a number of critical gene networks (Andrew et al. 2007), alter the vertebrate innate immune response (Nayak et al. 2007), and interfere with DNA repair processes (Andrew et al. 2006). Genomic profiling studies have reported that moderate changes in low-level exposure conditions elicited different expression profiles, suggesting arsenic affects biological systems at low levels, and these effects are very complex (Andrew et al. 2003).

In a human study in Argentina, Concha et al. (1998) found fetal arsenic levels similar to levels detected in mothers exposed via drinking water, indicating that arsenic crosses the placenta. Arsenic can affect placental vasculogenesis and increase the rate of spontaneous abortions (Andrew et al. 2006; He et al. 2007); cause epigenetic modifications (Xie et al. 2007); and induce neural tube defects, cause axial skeletal abnormalities, and reduce mean fetal weight in transplacentally exposed mice without evidence of maternal toxicity (Hill et al. 2008). Gene expression studies in exposed mouse fetal liver cells recapitulate those associated with transplacental arsenic-induced mouse liver tumors (Liu et al. 2008).

Early environmental challenges can affect disease susceptibility later in life in the absence of apparent alterations in normal developmental programs (Hales and Barker 2001; Newbold 2004; Newbold et al. 2006; Ravelli et al. 1998; Vickers et al. 2007). Diseases postulated to have developmental windows of vulnerability include cancers and neurologic, reproductive, and metabolic disorders (Birnbaum and Fenton 2003; Davey et al. 2007; Ho et al. 2006; Lahiri et al. 2007; Weidman et al. 2007). *In utero* exposure in mice was associated with dose-related adult-onset liver cancer, adrenal cortical adenoma in male offspring, and ovarian tumors and lung cancer in female offspring (Shen et al. 2007; Waalkes et al. 2007). The molecular basis of these correlations is largely unknown.

Emerging evidence from evolutionarily divergent species suggests that the immune response is compromised by low levels of arsenic and likely reflects functional disruption of critical genes and networks. An overrepresentation of genes involved in immunosuppression was correlated with arsenic exposure (Andrew et al. 2008), and arsenic down-regulated genes involved in immune modulation in mouse embryonic cells (Yu et al. 2008). Low levels of arsenic also reduced immune and defense responses in birds and fish (Aggarwal et al. 2008; Andrew et al. 2008; Yu et al. 2008). Additional studies in a tractable model are needed to better understand the extent to which arsenic perturbs regulatory networks involved in immune response during development.

The zebrafish (*Danio rerio*) is an ideal model for studying developmental toxicology and for understanding the connections between environmental exposures and human diseases (Pichler et al. 2003). Zebrafish are highly fecund with short generation times of 3–5 months. Eggs are fertilized externally and are relatively large (0.6 mm), transparent, and readily manipulated (Mattingly et al. 2001; Pichler et al. 2003). Rapid development from a zygote to the hatching period (~ 48 hr) provides advantages over mammalian models for observing organogenesis. Zebrafish are significantly more economical to maintain than are mammalian models, enabling more experimental opportunities (Pichler et al. 2003). Zebrafish resources are abundant, including a sequenced genome, microarray resources, and cDNA libraries. Despite the evolutionary distance separating zebrafish and humans (~ 400 million years), it is an important model of human diseases such as craniofacial, hematopoietic, cardiovascular, and neuro-degenerative disorders and cancer (Goessling et al. 2007; Lieschke and Currie 2007). Large-scale genetic screens have produced zebrafish mutants with phenotypes similar to human disorders (Goessling et al. 2007; Lieschke and Currie 2007). The combination of these features make the zebrafish uniquely suited for investigating the effects of environmental exposures and the consequences on vertebrate development.

In this study we aimed to identify genes and networks targeted by low levels of arsenic during vertebrate embryonic development. We exposed zebrafish embryos to arsenic; evaluated effects on transcription by whole genome micro array analysis, gene enrichment, and pathway analyses; and confirmed results by quantitative reverse transcriptase (RT) polymerase chain reaction (QPCR). Here we describe the effects of arsenic on the expression levels of a cohort of genes predicted to affect multiple vertebrate immune response processes.

## Materials and Methods

### Zebrafish

Wild-type AB zebrafish were maintained on a 14/10-hr light/dark cycle at the zebrafish facility of the University of Maine–Orono in a recirculating system with water at 28.5°C and a flow rate of 150 L/min. Animal husbandry was in accordance with the university’s Institutional Animal Care and Use Committee standards. Animals were treated humanely and with regard for alleviation of suffering.

### Arsenic exposures

Zebrafish adults were spawned; embryos were then collected, scored for viability, and sorted by developmental stage. We used embryos between 2-cell and 16-cell stage [0.25–1.5 hr postfertilization (hpf)] exclusively for the studies described here.

For microarray analysis, we exposed pooled samples of 50 zebrafish to 0, 10, or 100 ppb sodium *meta-*arsenite (NaAsO_2_; As^3+^; Fluka, St. Louis, MO) up to 48 hpf. These concentrations are environmentally relevant and do not cause toxicity. All exposures were in 0.5 × E2 media (7.5 mM NaCl, 0.25 mM KCl, 0.5 mM MgSO_4_, 0.075 mM KH_2_PO_4_, 0.0025 mM Na_2_HPO_4_, 0.5 mM CaCl_2_, and 0.35 mM NaHCO_3_). Embryos were kept at a density of 3–4/mL with one medium change per 24 hr. All exposures and controls were performed in triplicate in plastic Petri dishes at 28.5°C.

For QPCR analysis, we exposed pooled samples as described above, except that embryos were exposed at 24 hr. Four or five biological replicates were performed in plastic Petri dishes at 28.5°C.

### RNA extraction and reverse transcription

Total RNA was recovered from whole embryos using TRIzol (Invitrogen, Carlsbad, CA) according to the manufacturer’s protocol. We assessed RNA used for microarray and QPCR experiments by microchip analysis on an Agilent 2100 Bioanalyzer (Agilent Technologies, Foster City, CA). First-strand cDNA was synthesized from 2 mg of total RNA using Moloney murine leukemia virus RT from a RETROscript kit (Ambion, Austin, TX) according to the manufacturer’s protocol. Total RNA without RT were used as negative controls.

### Microarray analysis

We performed microarray analysis to evaluate the effects of arsenic exposure on global transcription during zebrafish development. Total RNA (~ 10 μg per biological replicate) was sent to the Affymetrix Core Facility of the Oregon Health and Sciences University (Portland OR), where microarray analyses were performed in triplicate on Affymetrix GeneChip Zebrafish Genome arrays consisting of 14,900 transcripts, for a total of nine independent arrays: three controls, three low- concentration As^3+^ (10 ppb) exposures, and three high-concentration As^3+^ (100 ppb) exposures.

#### Sample labeling

We converted RNA to double-stranded cDNA using Superscript Reverse Transcriptase (Invitrogen) and an oligo-dT primer linked to a T7 RNA polymerase binding site sequence. Amplified and labeled cRNA (“target”) was produced by *in vitro* transcription using T7 RNA polymerase, biotin-UTP, and biotin-CTP (Enzo Diagnostics, Inc., Farmingdale, NY). Target yield was measured by ultraviolet absorbance (λ_260_).

#### Array hybridization and processing

We fragmented labeled target at 95°C in the presence of high [Mg^2+^] and combined with biotinylated hybridization control oligomer and biotinylated control cRNAs for BioB, BioC, BioD, and CreX (Affymetrix) in hybridization buffer. We hybridized 10 μg of target with the arrays overnight, followed by washing, staining with streptavidin-phycoerythrin (Molecular Probes, Carlsbad, CA), signal amplification with biotinylated anti-streptavidin antibody (Vector Laboratories, Burlingame, CA), and a final staining step on the Fluidics Station 400 (Affymetrix). The distribution of fluorescent material on the processed array was determined using the Affymetrix GeneChip laser scanner; image inspection was performed manually. We created Affymetrix data files [cell intensity (CEL) files] from each array using GCOS (GeneChip Operating Software; Affymetrix).

#### Statistical analysis

We implemented a statisti cal process similar to the one reported by Gosse et al. (2008). Probe-level data from CEL files were normalized using robust multiarray analysis (Irizarry et al. 2003) as implemented in Bioconductor (http://www.bioconductor.org). Quality control was performed using log-ratio versus log-product (MA) plots and volcano plots (data not shown). Probes with a substantial likelihood of differential expression under treatment conditions were identified using simple *t*-tests combined with mean fold change in accordance with recommendations from the Microarray Quality Control (MAQC) Consortium (Guo et al. 2006; Shi et al. 2006). We selected a *t*-test *p*-value threshold of 0.1 and a minimum absolute fold difference of 1.4 between the controls and exposed data sets. This approach yielded 99 genes that were hierarchically clustered (Eisen et al. 1998), which we used in pathway analysis.

### Molecular pathway, gene ontology, and Comparative Toxicogenomics Database (CTD) analysis

To identify affected molecular pathways and biological processes, we computationally analyzed arsenic-responsive genes identified by microarray analysis. We identified human orthologs of affected zebrafish genes by deriving orthologs from Affymetrix annotations, by BLAST (Basic Local Alignment Search Tool) database [National Center for Biotechnology Information (NCBI) 2009] analysis of Affymetrix probe sequences (reciprocal and BLASTX) or by computational genome mapping. Orthologous human gene symbols were analyzed using Ingenuity Pathway Analysis (IPA) software (Ingenuity Systems, Redwood City, CA). Gene ontology assignments (Ashburner et al. 2000) and clustering into functional groups were performed using DAVID (Database for Annotation, Visualization, and Integrated Discovery) (Dennis et al. 2003). Microarray-derived gene expression changes were compared with manually curated arsenic–gene/protein interactions archived in CTD (CTD 2009; Davis et al. 2008, 2009). We used the CTD Batch Query tool (http://ctd.mdibl.org/tools/batchQuery.go) to retrieve all curated chemical–gene interactions for the terms “arsenic” and “arsenicals.”

### QPCR analysis

We performed QPCR analysis on an Mx3000P Real-Time PCR system (Stratagene, La Jolla, CA) using the Brilliant SYBR Green QPCR reagent kit (Stratagene) according to the manufacturer’s protocol. The cycling parameters were 95°C for 10 min, followed by 40 cycles of 95°C for 30 sec, 55°C for 60 sec, and 72°C for 60 sec. reshold cycles (C_t_) and dissociation curves were determined with MxPro software (Stratagene), and gene expression levels were normalized to zebrafish *Gapdh*.Standard curves and primer efficiencies were determined for all genes analyzed by QPCR. Primer sequences are listed in Table 1.

## Results

### Arsenic-mediated perturbation of gene transcription

**Figure 1:**
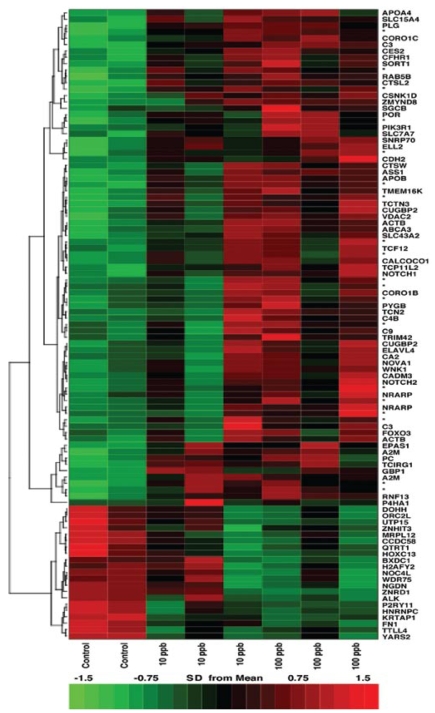
Hierarchical clustering of transcripts significantly modified by exposure to arsenic for 48 hr. Transcripts were selected by a fold-change ranking approach implemented in R (R Development Core Team 2009) using an absolute fold change of 1.4 and a threshold (*p*-value) of 0.1. Each column represents the expression level of a probe set in a pooled group of 50 animals (green and red indicate decreased and increased expression, respectively). Arrays are grouped by arsenic concentration [control (0 ppb), 10 ppb, and 100 ppb]. Transcripts were clustered by hierarchical clustering using the complete linkage algorithm and Pearson correlation metric in R.

We implemented several strategies during the statistical analysis of the microarray data sets. First, an uncorrected *t-*test identified-766 genes differentially expressed between control and treated samples (*p* < 0.05). However, data sets composed of > 15,600 measurements are expected to yield > 770 measurements by chance with *p*< 0 .05. After correcting for multiple hypothesis testing (Bonferroni correction), none of the genes rose to the level of significance. Other parametric tests performed similarly. Second, we implemented a nonparametric fold-change ranking approach as recommended by the MAQC Consortium (Guo et al. 2006; Shi et al. 2006) by which we selected genes with *a*) at least a ± 1.4-fold change between control and exposed data sets and *b*) substantial within-group consistency as evidenced by a two-tailed *t-*test- *p*-value ≤ 0.1. This approach yielded 99 differentially expressed genes, of which 55 had an uncorrected *p*-value ≤ 0.05. This refined data set was hierarchically clustered to produce the heat map shown in Figure 1, revealing a pattern suggesting concentration-dependent effects even though corrected *p-*values for this ranked set did not rise to the significance level of ≤ 0.05. Control (Figure 1, columns 1 and 2) and high-As^3+^–exposed (Figure 1, columns 6–8) biological replicates exhibited consistent reproducibility among replicates, whereas low-As^3+^–exposed replicates (Figure 1, columns 3–5) did not. For example, biological replicates 1 and 2 of the 10-ppb As^3+^ set (columns 3 and 4) were consistent with each other, whereas replicate 3 (column 5) exhibited a response in line with the biological replicates treated with 100-ppb As^3+^.

### Computational prediction of an arsenic-modulated molecular network affecting immune response

**Figure 2:**
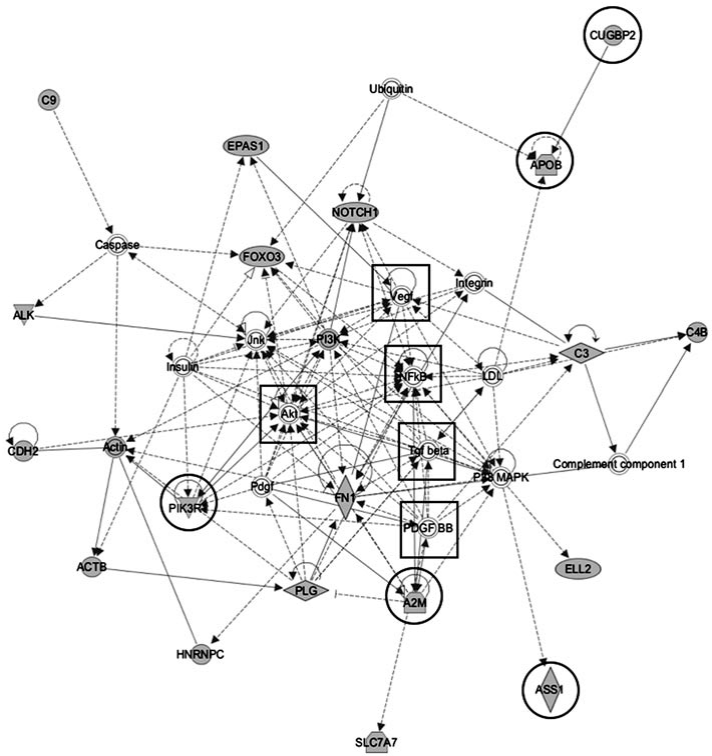
Predicted arsenic-modulated network. Differentially expressed genes in control versus arsenic-treated embryos were analyzed by IPA software to identify common regulatory networks. Nineteen genes identified by microarray analysis were shown to function coordinately in a network associated with cancer, gastrointestinal diseases, and immune response (*p* < 10^−41^), conditions known to be associated with arsenic exposure. Curated arsenic–gene and protein interactions in CTD corroborated 10 of the genes in this network—5 that derived from our microarray experiment (circled) and 5 that were inserted by IPA as bridging genes (boxed).

We used IPA to identify molecular relationships among genes predicted by microarray analysis to be differentially expressed in response to arsenic. In addition, noninput molecules are inserted by IPA in order to merge small networks generated from user data. We identified orthologous human genes for 79 of the 99 differentially expressed zebrafish genes described above. Among these, 64 were in the IPA knowledgebase. IPA identified a highly significant network (*p* < 10^−41^) containing 20 of the 64 (31%) input genes and 15 bridging genes (Figure 2). This network was significantly associated with immune response, cancer, and gastro intestinal disease (*p* ≤ 0.02). Genes within this network are involved in specific immune functions such as complement activation (*p* ≤ 2.8 × 10^−6^), migration of immune response cells (e.g., monocytes, macrophages; *p* < 9.2 × 10^−3^), and respiratory burst (*p* < 1.2 × 10^−2^). The IPA-derived network also contained three genes (*cugbp2*, *foxo5*, and *pik3r1*) predictive of prenatal arsenic exposure based on a recent epidemiologic study (Fry et al. 2007), two of which (*foxo5* and *pik3r1*) were observed in the 99 genes identified by the microarray analysis. These results support the hypothesis that the zebrafish is a valuable model organism for understanding the complex mechanisms of arsenic action during vertebrate development.

IPA results were corroborated by a gene ontology enrichment analysis conducted using the online Functional Annotation Clustering tool from DAVID (Dennis et al. 2003). Among the 79 differentially expressed genes with identifiable human orthologs, the “immune response” biological process [Gene Ontology (GO) ID no. GO:0006955 (The Gene Ontology 2009)] was significantly over-represented (*p* < 0.05). Analysis using the same 79 genes from the micro array analysis plus the 15 bridging genes from IPA yielded a subset of GO terms, 53% of which were associated with the immune system or defense responses (Table 2).

### CTD analysis

CTD contains a robust data set for arsenic that describes molecular interactions between 20 different arsenic compounds and 1,709 genes and proteins (Davis et al. 2008). Comparison of the 79 differentially expressed genes from the microarray analysis with curated arsenic-interacting genes in CTD yielded an over lapping set of 11 genes. Among these 11 genes, 5 were members of the predicted pathway (Figure 2, circled genes). CTD also corroborated 5 of the 15 bridging genes inserted by IPA (Figure 2, boxed genes). These genes were present on the microarrays but were not included in the top 99 genes identified by nonparametric rank-based analysis.

CTD also supported arsenic-associated GO terms and diseases identified by DAVID and IPA. The most common disease categories associated with arsenic in CTD include immune system diseases, neoplasms, nervous system diseases, skin diseases, digestive system diseases, and metabolic disorders (Davis et al. 2008). Results from IPA network analysis, microarray analysis, and data mining of CTD converged, reinforcing the potential effects of arsenic on this predicted network.

### Quantitative analysis of transcript levels of IPA network genes

**Figure 3:**
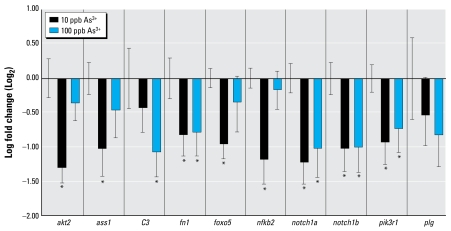
QPCR results for genes identified by microarray and CTD analysis to be affected by 24-hr exposure to As^3+^. Bars represent expression changes (mean fold change ± SD) compared with the corresponding control. With the exception of *ass1*, genes are involved in immune response. For each gene, initial error bars shown alone indicate control values *p < 0.05.

We analyzed a total of 10 genes within the network by QPCR: 6 derived from the micro array study (*C3*, *fn1*, *foxo5*, *notch1a*, *notch1b*, and *plg*; Figure 2); 2 derived from the microarray study and corroborated by CTD (*ass1*, *pik3r1*), and 2 bridging genes corroborated by CTD (*akt2*, *nfkb2*). We selected a 24-hr time point in order to analyze the effects of As^3+^ on genes potentially regulating the immune response during developmental stages earlier than the one analyzed by microarray. As shown in Figure 3 and Table 1, the genes selected for this analysis were down-regulated by As^3+^. Remarkably, this gene set exhibited a more robust and significant response to the lowest levels of As^3+^ (10 ppb) compared with responses to 10-fold higher levels of As^3+^ (9 of 11 genes vs. 6 of 11). Only 4 of the 11 genes tested showed similar responses to As^3+^ at both concentrations: *fn1*, *notch1a*, *notch1b*, and *pik3r1*. One gene, *plg*, had no expression change in control versus treated embryos, even though it was identified through microarray analysis to be affected by As^3+^.

**Figure 4:**
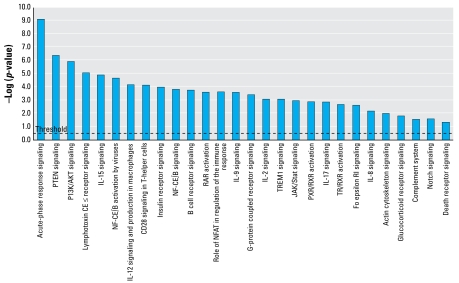
Canonical pathways identified by IPA associated with genes shown by QPCR to be arsenic responsive (threshold line indicates *p* < 0.05). These pathways are enriched with immune response functions. NFAT, nuclear factor of activated T cells.

We identified canonical pathways associated with these nine arsenic-responsive genes using IPA (Figure 4). Many of the pathways were involved in immune function, the most significant being acute-phase response signaling. Other pathways included lymphotoxin receptor signaling, interleukin signaling, CD28 signaling, Trem1 (triggering receptor expressed on myeloid cells 1) signaling, and the complement system. Collectively, gene ontology and pathway enrichment analyses of microarray and QPCR results demonstrate that arsenic perturbs genes and networks that are involved in the immune response during vertebrate development.

## Discussion

In this article we study the effect of As^3+^ exposure on the transcription profile of zebrafish embryos at 24 and 48 hpf by QPCR and microarray analysis, respectively. QPCR results demonstrate statistically significant down-regulation of multiple genes with critical functions in immune system development and, by inference, immunity at 24 hpf. In contrast, micro array analysis performed at 48 hpf reveals the opposite pattern: Genes down-regulated at 24 hpf are up-regulated at 48 hpf in the presence of As^3+^. The microarray analysis provided a robust target gene set that underwent changes in expression due to As^3+^ exposure, even though the set was below the threshold of significance as determined by statistical analysis. This observation underscores the need to take into account that low-dose exposures may not result in dramatic changes in gene expression that stand up to rigorous statistical tests but nevertheless contribute to significant biological effects that can be validated (Gosse et al. 2008).

The genes we evaluated by QPCR showed expression changes for at least one exposure concentration and in many cases for both. For example, *fn1*, *notch1a*, *notch1b*, and *pik3r1* were down-regulated at least 1.7-fold by both 10 and 100 ppb As^3+^ compared with controls, whereas *akt2*, *ass1*, and *nfkb2* responded significantly to 10 ppb As^3+^ (fold change ≥ 2) but not to 100 ppb As^3+^. Conversely, *C3* was down-regulated 2-fold by 100 ppb As^3+^ but showed no effect to 10 ppb As^3+^ exposures. This variable As^3+^ concentration-dependent phenomenon has been observed before. In a study by Bodwell et al. (2004), the effects of As^3+^ on activation of a reporter gene driven by a glucocorticoid-regulated promoter were stronger at low concentrations. In fact, they observed 75% of the maximal induction at 6 ppb As^3+^, with a peak at 60 ppb As^3+^, dropping off rapidly at higher concentrations to below basal levels of expression.

In the present study, IPA analysis identified a gene network containing 20 of 79 genes identified by microarray analysis (Figure 2). We found two genes, *foxo5* (the zebrafish ortholog of human *FOXO3A*) and *pik3r1*, in a group of 170 “sentinel” genes reported as predictive of transplacental As^3+^ exposure in infants (Fry et al. 2007). Additionally, bridging genes inserted into the network by IPA included *akt2*, *cugbp2*, *nfkb2*, and *pi3k*. The genes *akt2*, *nfkb2*, and *pi3k* are network hubs linking most genes within the set of 20 genes, and *akt2* and *pi3k* are members of the Akt/PI3K signal transduction axis important for insulin function, whereas *nfkb2* is required for immune processes (Speirs et al. 2004). Fry et al. (2007) identified *nfkb2* as one of two hub genes composing a subnetwork enriched with proinflammatory genes that were arsenic responsive. The concordance of arsenic-mediated effects in humans and zebrafish reinforces the immune system as a target of arsenic action and underscores the power of zebrafish as a vertebrate model for elucidating the effects of As^3+^ exposure during embryogenesis.

Epidemiologic studies have linked exposure to inorganic arsenic (As^3+^) to multiple human diseases (Hughes 2006). Several of these diseases, most notably cancer, might be rooted in an abnormal immune response. Arsenic is linked to disrupted expression of specific tumor suppressors by processes that alter promoter methylation patterns and lead to higher incidences of cancer, including bladder and lung, in exposed populations (Marsit et al. 2006). This finding, coupled to results demonstrating that As^3+^ interferes with DNA repair (Andrew et al. 2006), raises the possibility that arsenic may lead to transgenerational effects in unexposed individuals via epigenetic mechanisms. Studies demonstrate that very low-level exposures (in the range of 2–10 ppb) during zebrafish embryogenesis affect the innate immune system’s ability to respond to bacterial and viral pathogens at time points when the adaptive immune system is not fully developed (Nayak et al. 2007). Inorganic arsenic decreases levels of T-cell–secreted cytokines in exposed humans (Biswas et al. 2008); interferes with lung epithelial wound repair *in vivo* and *in vitro* (Olsen et al. 2008); drastically down-regulates expression levels of genes required for B-cell antigen recognition, humoral immune response, and antigen binding (Andrew et al. 2007a); inhibits monocyte-to-macrophage maturation *in vitro* (Sakurai et al. 2006); and decreases proliferation of CD4^+^/CD8^+^ T lymphocytes *in vitro* (Tenorio and Saavedra 2005). In addition, immune-related inflammation disorders or activation of inflammation signaling pathways have been observed in humans and rodents chronically exposed to As^3+^ (Fry et al. 2007; Straub et al. 2007; Vahidnia et al. 2007; Wu et al. 2003). These results establish a link between As^3+^ exposure and immune disorders, yet with the exception of work by Nayak et al. (2007), they do not indicate whether the effects are restricted to disrupting the function of specific populations of differentiated cells or if exposure at early stages of development leads to abnormal immune responses. Nayak et al. (2007) measured a significant decrease in expression of several immune system modulators, including members of the interleukin family, in juvenile fish previously exposed to low levels of As^3+^. However, insight into arsenic-mediated effects on the embryonic transcriptome requires more attention in order to gain greater insight into the effects of arsenic on the immune system and associated disorders.

## Conclusion

This study shows that the zebrafish is a valuable model organism to enhance understanding of the effects of arsenic on aspects of embryology, including networks affecting immune development. The networked genes uncovered by this study are highly evolutionarily conserved at various levels, including conservation of chromosomal synteny (data not shown). In addition, there is significant overlap between the results of this study and observations made by others. These data enhance understanding about developmental responses induced by arsenic and provide novel insight into the molecular actions of arsenic on the immune system.

## Figures and Tables

**Table 1:** Genes and primers analyzed by QPCR.

Gene symbol (name)^b^	Fold change^a^		Gene accession ID	Primer sequence	Amplicon (bp)
	10	100			
*akt2* (v-akt murine thymoma viral oncogene homolog 2)	−2.4	NC	NM_198146	5′-GAGATCAGCGTCGTCAGAGA-3′ (F)	
				5′-AGCCGATAAAAGAGCCATCA-3′ (R)	106
*ass1* (argininosuccinate synthetase 1)	−2.0	NC	NM_001004603	5′-GGAGGATCGATATCGTGGAG-3′ (F)	
				5′-GTCCAGATGAGCCTGAAGGA-3′ (R)	127
*C3* (similar to complement C3)	NC	−2.0	XM_001343352	5′-GCTGTGCACGTCCTTAACAA-3′ (F)	
				5′-CATCTCTTCCACCTCCTGCT-3′ (R)	103
*fn1* (fibronectin 1)	−1.8	−1.7	NM_131520	5′-TGCGGCACGACTTATAACTTT-3′ (F)	
				5′-TCACACCCTCATTGGTGGTA-3′ (R)	94
*foxo5* (forkhead box O5)	−1.9	NC	NM_131085	5′-TGAATGGGAGGAGAGGTGTT-3′ (F)	
				5′-GTCACATTCGCATTCCATGA-3′ (R)	100
*gapdh* (glyceraldehyde-3-phosphate dehydrogenase)	Norm	Norm	NM_001115114	5′-TGGGCCCATGAAAGGAAT-3′ (F)	
				5′-ACCAGCGTCAAAGATGGATG-3′ (R)	94
*nfkb2* (nuclear factor of kappa light polypeptide gene enhancer in B-cells 2)	−2.3	NC	NM_001001840	5′-TGAATGGGAGGCATTAGGAG-3′ (F)	
				5′-ACAGGTCGATCGATGTTGGT-3′ (R)	105
*notch1a* (notch homolog 1a)	−2.3	−2.0	NM_131441	5′-TGACGTTAACGAGTGCCTGT-3′ (F)	
				5′-GCTTCCCGGTGTATCCTGTA-3′ (R)	110
*notch1b* (notch homolog 1b)	−2.0	−2.0	NM_131302	5′-ATTGATGATGTGGCCGGATA-3′ (F)	
				5′-TCATTTCGGCAAGGATTTTT-3′ (R)	114
*pik3r1* (similar to phosphoinositide-3-kinase, regulatory subunit, polypeptide 1)	−1.9	−1.7	XM_678727	5′-GATGATACGCATCGCTCAAG-3′ (F)	
				5′-TGTGGAGGAAGTGCAGTTGA-3′ (R)	90
*plg* (plasminogen)	NC	NC	NM_201472	5′-ATGGAGCCTCATCGACATTC-3′ (F)	
				5′-TAACACCAAGGGGCTCTGTC-3′ (R)	104

Abbreviations: F, forward primer; NC, no change; Norm, normalizer; R, reverse primer.

a Fold change in gene expression between As^3+^ (10 or 100 ppb) and control (0 ppb) experiments as determined by QPCR analysis. FC = −1/(2^ΔΔCt^), where ΔΔC_t_ = ΔC_t,exposed_ − ΔC_t,control_. ΔC_t,exposed_ and ΔC_t,control_ are the normalized threshold cycles for exposed and control samples, respectively.

b Official zebrafish gene symbols and gene accession numbers from NCBI (2009).

**Table 2:** Immune processes enriched with genes perturbed by arsenic.

GO ID^a^	GO process	Gene count	*p*-Value	Human genes^b^
GO:0002253	Activation of immune response	4	3.97 × 10^−3^	*C3*, *C4B*, *C9*, *CFHR1*
GO:0006952	Defense response	8	1.81 × 10^−2^	*C3*, *C4B*, *C9*, *CFHR1*, *FN1, INS*, *P2RY11*, *TCIRG1*
GO:0009605	Response to external stimulus	8	3.07 × 10^−2^	*A2M*, *C3*, *C4B*, *C9*, *CFHR1, FN1*, *INS*, *PLG*
GO:0006950	Response to stress	10	6.45 × 10^−2^	*APOA4*, *C3*, *C4B*, *C9, CFHR1*, *CSNK1D*, *EPAS1, FN1*, *INS*, *PLG*
GO:0006955	Immune response	9	7.12 × 10^−2^	*C3*, *C9*, *C4B*, *CFHR1, CTSW*, *GBP1*, *NFKB2, NOTCH1*, *TCF12*
GO:0002520	Immune system development	4	8.25 × 10^−2^	*FOXO3*, *NFKB2*, *NOTCH2, PIK3R1*

a Data from The Gene Ontology (2009).

b Data from NCBI (2009).
